# *De Novo* Assembly and Characterization of the Transcriptome of Seagrass *Zostera marina* Using Illumina Paired-End Sequencing

**DOI:** 10.1371/journal.pone.0112245

**Published:** 2014-11-25

**Authors:** Fanna Kong, Hong Li, Peipei Sun, Yang Zhou, Yunxiang Mao

**Affiliations:** Key Laboratory of Marine Genetics and Breeding, College of Marine Life Sciences, Ocean University of China, Qingdao, China; Università della Calabria, Italy

## Abstract

**Background:**

The seagrass *Zostera marina* is a monocotyledonous angiosperm belonging to a polyphyletic group of plants that can live submerged in marine habitats. *Zostera marina* L. is one of the most common seagrasses and is considered a cornerstone of marine plant molecular ecology research and comparative studies. However, the mechanisms underlying its adaptation to the marine environment still remain poorly understood due to limited transcriptomic and genomic data.

**Principal Findings:**

Here we explored the transcriptome of *Z. marina* leaves under different environmental conditions using Illumina paired-end sequencing. Approximately 55 million sequencing reads were obtained, representing 58,457 transcripts that correspond to 24,216 unigenes. A total of 14,389 (59.41%) unigenes were annotated by blast searches against the NCBI non-redundant protein database. 45.18% and 46.91% of the unigenes had significant similarity with proteins in the Swiss-Prot database and Pfam database, respectively. Among these, 13,897 unigenes were assigned to 57 Gene Ontology (GO) terms and 4,745 unigenes were identified and mapped to 233 pathways via functional annotation against the Kyoto Encyclopedia of Genes and Genomes pathway database (KEGG). We compared the orthologous gene family of the *Z. marina* transcriptome to *Oryza sativa* and *Pyropia yezoensis* and 11,667 orthologous gene families are specific to *Z. marina*. Furthermore, we identified the photoreceptors sensing red/far-red light and blue light. Also, we identified a large number of genes that are involved in ion transporters and channels including Na^+^ efflux, K^+^ uptake, Cl^−^ channels, and H^+^ pumping.

**Conclusions:**

Our study contains an extensive sequencing and gene-annotation analysis of *Z. marina*. This information represents a genetic resource for the discovery of genes related to light sensing and salt tolerance in this species. Our transcriptome can be further utilized in future studies on molecular adaptation to abiotic stress in *Z. marina*.

## Introduction

Seagrasses are a polyphyletic group of plants that can live submerged in marine habitats [1]. The group consists of 60 monocotyledonous angiosperm species. In recent years, ecologists have focused on the ecological roles of seagrasses and evolutionary biologists have paid more attention to their evolutionary significance. Seagrasses are believed to have returned to the sea through at least three independent parallel-evolution events, evolving from a common freshwater ancestor of terrestrial origin [2],[2,3]. During the transition to a marine environment, seagrasses would have had to incorporate morphological and physiological mechanisms distinct from terrestrial angiosperms. These changes include morphologies, life history strategies, and breeding systems [3]. Specific to the marine environment, seagrasses also evolved the ability to tolerate high salt levels, short-term salinity fluctuations, low-intensity light, and a different light spectrum from that encountered by terrestrial angiosperms. These novel adaptations certainly involved specific genetic mechanisms, so developing the genes at a high throughput level will help generate an understanding of the adaptation mechanisms, evolutionary process, and parallel gene transfer events of seagrass.

*Zostera marina* L. belongs to the family *Zosteraceae*, and is one of the most common seagrasses. This seagrass is distributed mainly in temperate regions of the northern and southern hemispheres. *Z. marina* is an important marine primary producer that provides food and shelter for marine organisms. With the development of the genome sequencing project at the Joint Genome Institute (http://www.jgi.doe.gov/), *Z. marina* was believed to be the cornerstone of marine plant molecular ecology research and comparative studies [4]. Global warming is a serious threat to the growth and maintenance of the global *Z. marina* populations. Using high-throughput transcriptome sequencing, several studies have investigated the transcriptomic profiles of the plant under different temperature stress treatments. These studies provided an important foundation for predicting how *Z. marina* will respond to the increasing climatic extremes predicted under global warming [3]–[6]. Kong obtained a *Z. marina* transcript profile by sequencing a non-normalized cDNA library from a salinity treatment and identified a number of salt-tolerance genes. This provided insight into the molecular mechanisms of saline tolerance in *Z. marina* [7]. Furthermore, Wissler compared the transcriptome profiles of two representative seagrass species, *Z. marina* and *Posidonia oceanica*, with other terrestrial plants. The results showed that although total expression of ortholog genes between the two species was significantly similar, some orthologs still showed significant differences. These differences reflect both the degree of independent adaptation by the same mechanisms and non-parallel, lineage-specific adaptation to similar habitats [3],[4]. The above literature provides a foundation for understanding the adaptation mechanisms of *Z. marina* in response to the marine environment.

Many studies have focused on the physiological responses of *Z. marina* to different environmental stressors, including light and salinity. The majority of seagrass loss was considered to be light limitation, caused by human disturbance [8]. It is well known and accepted that under low light, total chlorophyll increased and the chlorophyll a∶ b ratio decreased, accompanied by sharp reductions in photosynthetic efficiencies and high photochemical efficiencies [9]–[11]. Seagrasses have been shown to exhibit a variety of mechanisms for acclimating to salinity fluctuations that range from changes in the cellular ion concentrations (organic osmolytes) to the elasticity of the cell wall [12]. Salinity changes of short-term and long-term can result in different physiological responses of *Zostera*. The short-term response involved osmotic adjustments of inorganic ions and organic osmolytes such as proline, carbohydrates, and organic acids [12]. Short term salinity changes also decreased quantum yields, but the seagrass can physiologically adjust after several days of chronic exposure [13]. Over the long term, salinity exerted significant effects on light-saturated photosynthetic rates (*P*max) of *Z. japonica* [14]. Furthermore, some reports showed that light, temperature and salinity influence seagrass physiological parameters such as C fixation, respiration and cellular osmotic pressure [15]. Detailed analyses of genes involved in the response of *Z. marina* to different stressors at the transcriptomic level remain scarce.

The recent advances in next-generation sequencing (NGS) technologies have shown great potential for expanding transcriptome databases for non-model organisms [16]. Compared to Roche 454, the Illumina Genome Analyzer has the limitation of short sequence reads. However, the improvements to read length via paired-end sequencing, and the development of bioinformatics and computational methods, have reduced both the cost and time required to generate gene expression profiles. The Illumina Genome Analyzer has been successfully used for non-model organisms in comparative transcriptomic studies to identify genes differentially expressed among different cultivars, organs, and treatment conditions [17]–[23]. Aside from gene discovery, many studies have demonstrated that transcriptome sequencing also represents an efficient way to address evolutionary questions and ecological gene expression variation [24]–[27].

In this research, we used the Illumina HiSeqTM 2000 platform to obtain a comprehensive transcriptome dataset of *Z. marina* under different stress factors (light intensity, temperature, pH, salinity, and light quality). We identified a series of unigenes related to osmotic regulation and photoreception, which provided a foundation to investigate the adaptation mechanisms of *Z. marina* for the marine environment. Furthermore, we compared the transcriptomes of *Z. marina*, *O. sativa*, and *P. yezoensis* to elucidate the functional and evolutionary processes that act on their respective functional genes.

## Materials and Methods

### Plant Materials and Experimental Treatment

*Z. marina* used in this study was collected in April 2013 from Huiquan Bay (Yellow Sea, 36°03′N, 120°20′E) in Qingdao, Shandong Province, China. The field study was permitted and supported by The Key Science and Technology Program of Shandong Province (Grant No. 2012GHY11527) and the Public Science and Technology Research Funds Projects of Ocean, State Oceanic Administration of China (Grant No. 201105021). This sea area is not privately owned and we didn't collect any protected species except seagrass. Plants were carefully removed by hand to ensure that their rhizome systems remained intact. They were then transplanted to the laboratory at the Ocean University of China (Shandong, China) where they were grown in glass tanks with 10 L of seawater (33 P.S.U. (practical salinity)) and 10 cm of sand. The tanks were kept at 10°C and exposed to 200 µmol photons·m^−2^·s^−1^ on a 16-h light/8-h dark cycle. The seawater was aerated continuously with filter-sterilized air and was renewed every three days. After two weeks, the plants were subjected to five sets of different environmental factors including temperature, salinity, light intensity, light quality, and pH value (Table 1). After 6 h, young leaves were collected and stored at −80°C until RNA extraction.

**Table 1:** Summary of different environmental stressors designed in this research.

Experimental sets	Temperature (°C)	Salinity	pH	Light intensity (µmol·m^−2^·s^−1^)	Light quality
**1**	4	33	8	125	sunlight
	28	33	8	125	sunlight
**2**	10	18	8	125	sunlight
	10	62	8	125	sunlight
**3**	10	33	6.2	125	sunlight
	10	33	8.7	125	sunlight
**4**	10	33	8	1.66	sunlight
	10	33	8	170	sunlight
**5**	10	33	8	125	Red light
	10	33	8	125	Blue light
	10	33	8	125	Green light

### RNA Extraction

Total RNA was extracted using the Plant RNA Kit (Omega, GA, USA) according to the manufacturer's instructions. RNase-free DNase I (Qiagen, Beijing, China) was used to remove residual gDNA via on-column digest. Total RNA purity and concentration were measured using the NanoPhotometer R spectrophotometer (Implen, CA, USA) and the Qubit RNA Assay Kit of the Qubit 2.0 Flurometer (Life Technologies, NY, USA), respectively. The RNA integrity was evaluated using the Agilent 2100 Bioanalyzer (Agilent Technologies, CA, USA) with a minimum RNA integrity number value of 8.

### Preparation of cDNA library and sequencing

Total RNAs for five different stressors were equally pooled for the construction of cDNA libraries. Sequencing libraries were generated using the Illumina TruSeqTM RNA Sample Preparation Kit (Illumina Inc, CA, USA) following the manufacturer's recommendations. The purification of mRNA was performed using poly-T oligo-attached magnetic beads. Fragmentation was carried out using divalent cations under elevated temperature in the Illumina proprietary fragmentation buffer. The cleaved RNA fragments were reverse transcribed into first strand cDNA using random oligonucleotides and SuperScript II. After second strand cDNA synthesis, fragments were end repaired, a-tailed, and indexed adapters were ligated. The products were purified and enriched by PCR to generate the final library. The library preparations were sequenced on an Illumina Hiseq 2000 platform to generate 100 bp paired-end reads.

### *De novo* transcriptome assembly

Transcriptome *de novo* assembly was carried out using the short read assembly program “Trinity” [28]. Raw data were scanned using the Casava software and low-quality reads were removed. The high-quality reads were loaded into the computer, and then a de Bruijn graph data structure was used to represent the overlap among the reads. The clean reads were deposited in the NCBI Sequence Read Archive (SRA) Database with accession number SRP035489.

### Transcriptome annotation

After *de novo* assembly with Trinity, the assembled unigenes were used for BLAST searches and annotation against the NCBI Nr database (NCBI non-redundant sequence database) using an E-value cut-off of 10^−5^. Unigene sequences were also aligned by BLASTX to protein databases such as Swiss-Prot and COG in order to retrieve proteins with the highest sequence similarity to the given unigenes along with their putative functional annotations. Based on the BLAST hits against the Nr and Swiss-Prot databases, GO (Gene Ontology) annotation was performed using the Blast2go program to obtain molecular function, biological process, and cellular component terms [29]. In order to obtain gene pathway networks, KEGG annotation was performed to identify the metabolic pathways of each KEGG orthology (KO) [30] number from each unigene using the online KEGG Automatic Annotation Server (KAAS) (http://www.genome.jp/kegg/kaas/) [31].

### Comparative analysis of Illumina sequencing with public data of other species

In order to verify the depth and coverage of the transcriptome data of the Illumina sequencing, Roche 454 Titanium reads (Accession no. SRP007220 SRA002573) and the NCBI EST database (Taxonomy ID: 29,655) were used as reference data. The Newbler 2.5 software package (Roche, Basel, Switzerland) was used for assembling, with the “extend low depth overlaps” parameter. Furthermore, we also compared the unigenes of *Z. marina* with those of *P. yezoensis* and the terrestrial monocotyledon *O. sativa* using the orthoMCL program to obtain their orthologous gene families [32]. *P. yezoensis* genes were from an Illumina sequencing transcriptome (Accession number SRP035567) and the *O. sativa* genes were collected from http://rapdb.dna.affrc.go.jp/download/irgsp1.html. A Venn diagram showing the distribution of shared and specific gene families among the three species was generated using an online Venn Diagram program [25] [33]. GO clusters among the three species were analyzed to compare their orthologies and to discover similarities and differences in evolutionary adaptation [29].

## Results

### Illumina Sequencing Output and *de novo* Assembly

To obtain a more comprehensive understanding of the functional genes from *Z. marina* adapted to different environment factors, we used the Illumina platform to sequence a pooled RNA sample covering the five abiotic stresses of temperature, salinity, light intensity, light quality, and pH. After a trimming process that removed low-quality reads, a total of 54,713,926 (98.54%) paired-end reads with an average length of 100 bp were generated from 55,525,824 raw reads. The final assembly generated a total of 5.48G base pairs with 98.5% of Q20, which represented 58,457 transcripts with an average length of 1,544 bp and an N50 length of 2,300 bp. This *de novo* assembly yielded 24,216 unigenes with an N50 of 1,802 bp. The length of unigenes ranged from 201 bp to 15,756 bp with an average of 1,006 bp (Table 2).

**Table 2:** Overview of sequencing and assembly results for *Z. marina*.

Sequencing results		Assembly results		
		Transcripts unigenes		
**Total number of raw reads**	55,525,824	**Number**	58,457	24,216
**Total number of clean reads**	54,713,926	**N50 (bp)**	2,300	1,802
**Average read length (bp)**	100	**Min length (bp)**	201	201
**Clean bases (G)**	5.48	**Mean length (bp)**	1,544	1,006
**Q20 (%)**	98.50	**Max length (bp)**	15,756	15,756
**GC (%)**	45.45			

The Illumina sequencing data were compared with Roche 454 pyrosequencing data and publicly available EST data, respectively (Table 3). A total of 58,457 transcripts from the Illumina sequencing were bi-blasted against 17,960 isotigs from the 454 Pyrosequencing. A total of 42,504 transcripts were matched to isotigs, accounting for 72.71% of total transcripts. There were still 27.29% transcripts not matched to isotigs. In parallel, 16,462 isotigs had hits in Illumina transcripts, accounting for 91.66% of total isotigs. In addition, the N50 length of the 454 sequencing was 1,407 bp, shorter than that of the Illumina sequencing. A total of 9,753 ESTs were matched to Illumina transcripts, accounting for 91.5% of total ESTs. These results indicate that the quality of our assembly was high and the coverage of our transcriptome transcripts was wide. This comparison also indicated that these transcripts can be complementary to transcriptome data in the public domain and can be used to enrich the existing expressed transcripts of *Z. marina*.

**Table 3:** Comparison of transcripts detected using Illumina sequencing with the publicly available transciptome and ESTs.

		A to B Blast hits/total number of queries	B to A Blast hits/total number of queries
A	B	(Blast hit rate)	(Blast hit rate)
**Illumina transcripts**	**454 Isotigs**	42,504/58,457	16,462/17,960
		(72.71%)	(91.66%)
**Illumina transcripts**	**EST**	13,488/58,457	9,753/10,659
		(23.07%)	(91.50%)

### Functional Annotation and Classification of assembled unigenes

**Figure 1:**
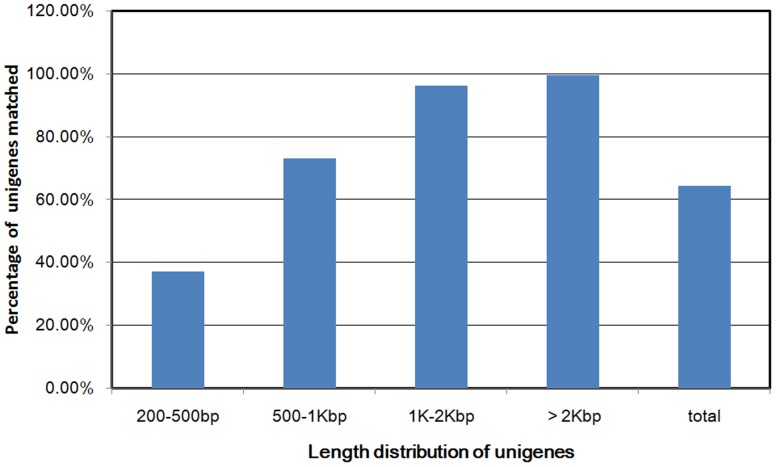
Comparison of unigene length between hit and no hit unigenes. Longer unigenes were more likely to have BLASTx homologs in the protein database.

In order to assess and annotate assembled unigenes, the 24,216 unigenes were subjected to BLASTx similarity analysis (E-value cutoff of 10^−5^) against different public databases including the NCBI non-redundant protein (Nr) database, the Swiss-Prot protein database, and the Nt database. The results showed that 15,571 (64.3%) of the 24,216 unigenes were significantly matched to known proteins in the public databases. There were 8,645 (35.7%) unigenes with unknown function, which could be specific to *Z. marina*. A total of 14,389 unigenes (59.41%) showed significant similarity to known proteins in the Nr database, and 10,941 (45.18%) had BLAST hits in the Swiss-prot database with an E value of 10^−5^. The mapping rates of unigenes against the Nt and Pfam databases were 16.29% and 46.91%, respectively (Table 4). Further analysis indicated that the longer the unigene, the higher the match efficiency. Match efficiencies of the unigenes with lengths longer than 2,000 bp and 1,000–2,000 bp were 99.68% and 96.30%, respectively. In contrast, for unigenes with the length of 200–500 bp, the annotation frequency across the databases was only 37.12% (Figure 1).

**Table 4:** Functional annotation of the *Z. marina* transcriptome.

	Number of unigenes	Percentage
**Annotated in NR**	14,389	59.41%
**Annotated in NT**	3,946	16.29%
**Annotated in KEGG**	4,745	19.59%
**Annotated in Swiss-Prot**	10,941	45.18%
**Annotated in Pfam**	11,362	46.91%
**Annotated in GO**	13,897	57.38%
**Annotated in COG**	7,195	29.71%
**Annotated in all databases**	1,604	6.62%
**Annotated in at least one database**	15,571	64.3%
**Total queries/unigenes**	24,216	100%

Distribution analysis based on BLASTx searches showed that the unigenes of *Z. marina* include homologs of function-known genes that are present in other plant species. Among the various species, the top six are listed in Table 5. Although *Z. marina* belongs to the monocotyledons, the top two species were *Glycine max* and *Vitis vinifera* with hit rates of 6.7% and 6.6%, respectively. The monocotyledon species *O. sativa* and *Zea mays* ranked fourth and fifth and had hit rates of 5.5% and 5.4%, respectively.

**Table 5:** Distribution of matched proteins in the NR database for the top six species.

Species	Number of unigenes	Total unigenes	Percentage
***G. max***	1617	24,216	6.7%
***V. vinifera***	1590	24,216	6.6%
***A. thaliana***	1422	24,216	5.9%
***O. sativa***	1324	24,216	5.5%
***Z. mays***	1308	24,216	5.4%
***P. trichocarpa***	1030	24,216	4.3%

**Figure 2:**
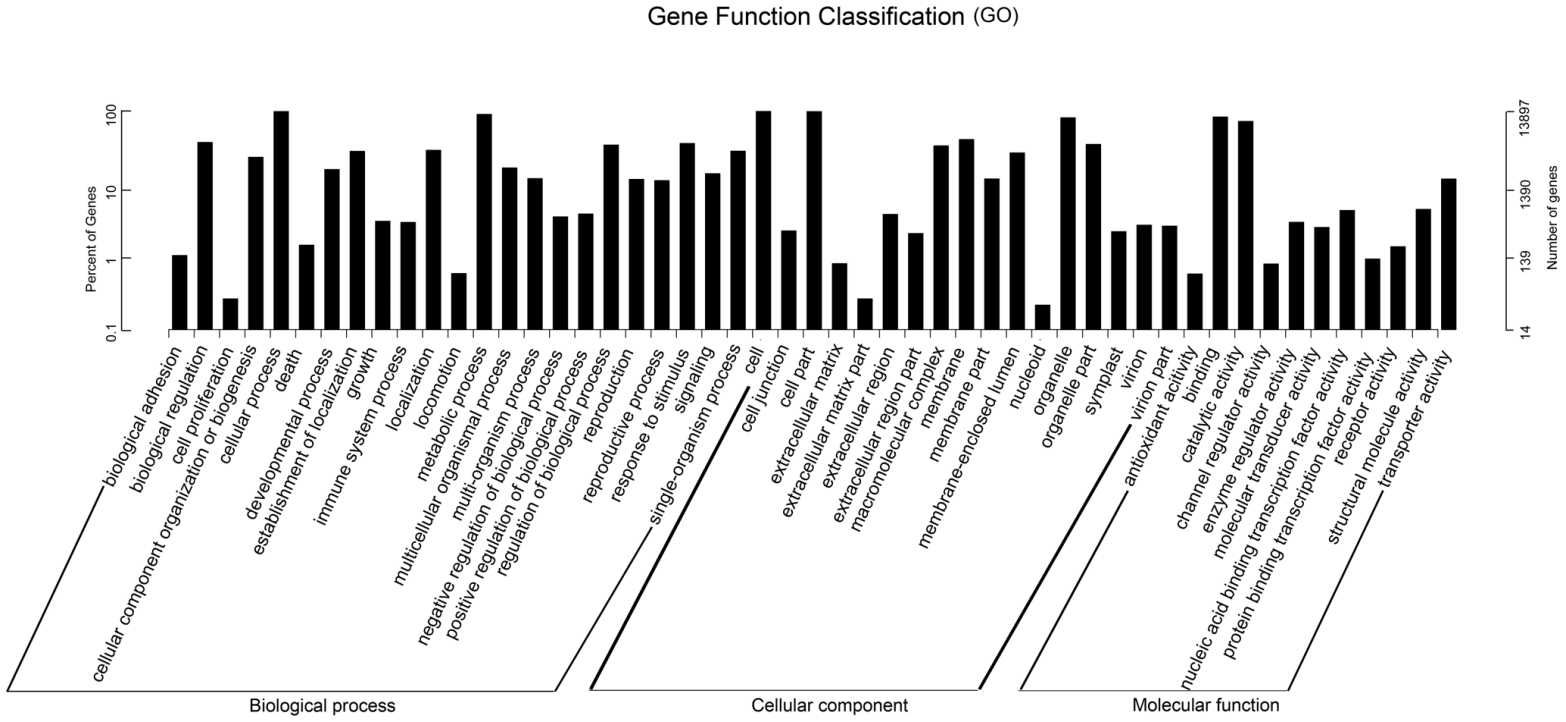
Gene Ontology (GO) function analysis for the *Z. marina* transcriptome. Totally 13,897 unigenes were assigned into three ontologies with 57 functional terms. Left Y-axis represents percentage of a specific category of genes in each main category. Right Y-axis indicates number of genes in a category.

In total, 13,897 (57.38%) unigenes were assigned into 57 level-2 GO terms with 116,120 terms that were distributed in three main ontologies including biological processes, cellular components, and molecular function. Each unigene was assigned to one or several GO Slims. Among them, the unigenes for biological process made up the majority (52,151, 44.9%) followed by the unigenes for cellular components (45,091, 38.8%) and those for molecular function (18,968, 16.3%) (Figure 2). The functionally assigned unigenes covered a comprehensive range of GO categories. Within biological processes, cellular process (9,760 unigenes, 18.71%) and metabolic process (8,973 unigenes, 17.21%) were the most abundant GO Slims which indicated that the *Z. marina* leaf tissues were actively performing extensive cellular and metabolic processes. In addition, 3,742 unigenes (7.2%) were involved in responses to different stimuli and 1,508 unigenes were related to signal transduction. In the cellular component ontology, cell (9,797 unigenes, 21.73%), cell part (9,742, 21, 61%), and organelle (8,093 unigenes, 17.95%) were prominently represented. In the molecular function ontology, the binding, catalytic activity, transporter activity, and structural molecule activity were the most highly represented groups (91.45%).

**Figure 3:**
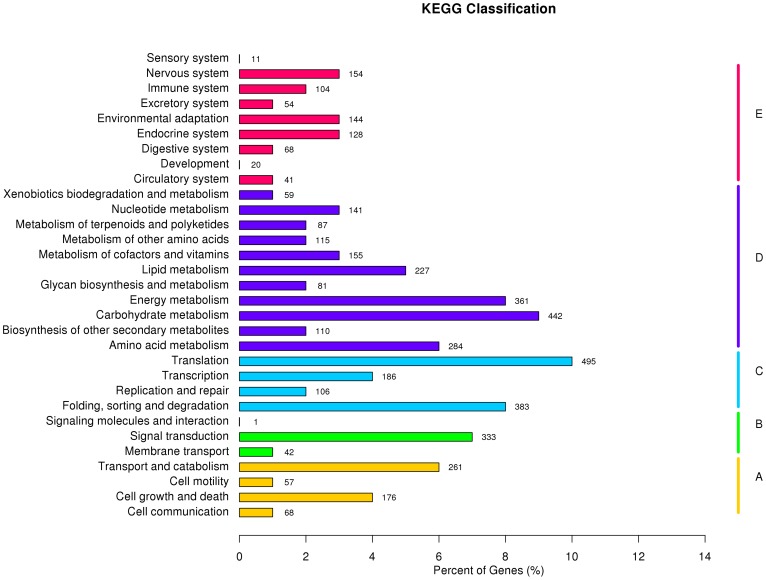
Pathway assignment based on KEGG for the *Z. marina* transcriptome. In total, 4,745 unigenes with Nr hits were grouped into 233 pathways.

Unigenes were compared with KEGG using BLASTx with an E-value of less than 10^−10^ and the corresponding pathways were established. A total of 4,745 unigenes were assigned to five main categories including 233 KEGG metabolic pathways (Table S1). Among the 5 main categories, metabolism was the biggest category (2,752, 43.09%) followed by genetic information processing (1,315, 20.59%), organismal systems (998, 15.63%), cellular processes (751, 11.76%), and environmental information processing (570, 8.93%) (Figure 3). These results provide further indication that active metabolic processes were underway in the *Z. marina* leaf tissues. Among 233 pathways, the pathways most strongly represented by mapped unigenes were “ribosome (ko03010),” “protein processing in endoplasmic reticulum (ko04141),” “spliceosome (ko03040),” and RNA transport (ko03013). Within the “metabolism category,” the largest pathway was “oxidative phosphorylation (ko00190)” and 113 unigenes were assigned. Particular attention was paid to the environmental adaptation. The top ranked pathway was “plant hormone signal transduction (ko04075)” and contained 95 unigenes followed by “PI3K-Akt signaling pathway (ko04151)” (67 unigenes), “phosphatidylinositol signaling system (ko04070)” (63 unigenes), and “Wnt signaling pathway (ko04310)” (61 unigenes). This suggests that phosphorylation and signal transduction are general processes regulating *Z. marina* adaptation to different environmental conditions. It is noteworthy that 11 unigenes were assigned to the “phototransduction pathway” (ko04744) of the sensory system.

### Comparative analysis of orthologous gene family

**Figure 4:**
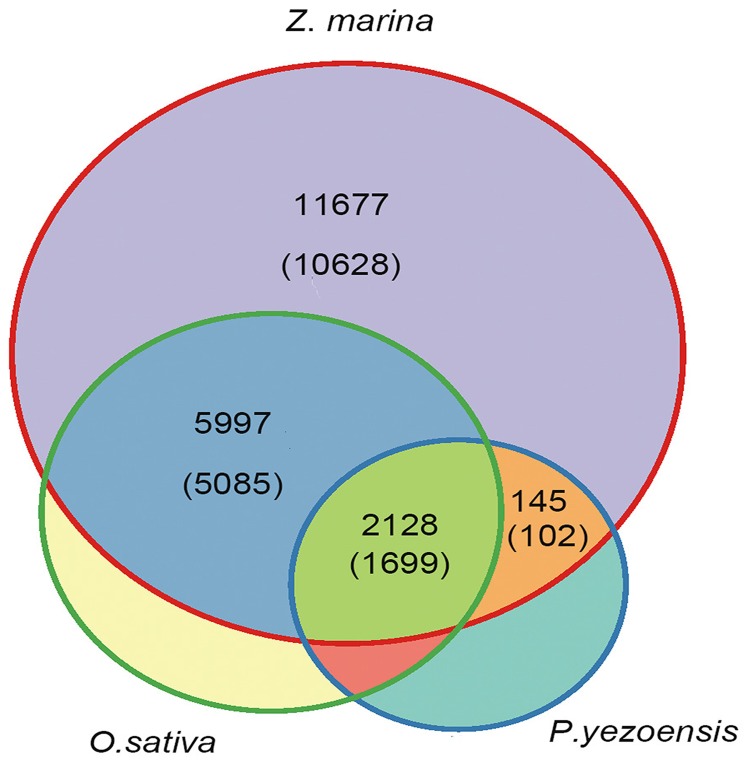
Venn diagram of ortholog group distribution in *Z. marina*, *P. yezoensis* and *O. sativa*. Numbers shown in different sections indicate the numbers of ortholog groups and unigenes of *Z. marina*. The top numbers give the number of unigenes and the bottom numbers give the number of ortholog groups.

In order to understand gene development during the *Z. marina* migration to a marine environment, we compared the ortholog groups of the *Z. marina* transcriptome to *O. sativa* and *P. yezoensis*. The orthology analysis was performed among 24,216 (*Z. marina*), 35,679 (*O. sativa*), and 18,640 (*P. yezoensis*) unigenes using orthoMCL [32]. A total of 10,616 ortholog groups were identified, of which, 7,204 were discovered in *Z. marina*, 9,670 in *O. sativa*, and 2,558 in *P. yezoensis*. These ortholog groups referred to 9,635 (*Z. marina*), 22,645 (*O. sativa*), and 4,275 (*P. yezoensis*) unigenes. There are 10,312 unigenes of *Z. marina* without ungrouped ortholog. The ortholog groups and unigenes of *Z. marina* were further analyzed. 1,699 ortholog groups (2,128 unigenes of *Z. marina*) were found to have reciprocal hits in all three pair-wise comparisons and were considered to be orthologs across *Z. marina*, *O. sativa*, and *P. yezoensis*. However, 5,085 orthologs (5,997 unigenes) had the best reciprocal hits between *Z. marina* and *O. sativa*. Only 102 orthologs (145 unigenes) occurred between *Z. marina* and *P. yezoensis*. It was worth noting that 316 orthologs (1,365 unigenes) only existed in *Z. marina*. Thus 11,677 orthologs were specific to *Z. marina*, plus 10,312 unigenes without ungrouped orthologs (Figure 4).

Functional analysis showed that the distribution patterns of the GO categories were more similar between *Z. marina* and *O. sativa* than between *Z. marina* and *P. yezoensis*. This indicates that the transcriptomic profile of *Z. marina* is more similar to *O. sativa* than to *P. yezoensis*, which is consistent with their species classification. Some Go Slim terms were specifically assigned to *Z. marina* and *O. sativa* such as “locomotion (GO: 0040011)” and “rhythmic process (GO:0048511)” in the biological process category, “nucleoid (GO:0009295)” in the cellular component category; and “antioxidant activity (GO:0016209),” “protein binding transcription factor activity (GO:0000988),” and “receptor activity (GO:0004872)” in the molecular function category (Figure 5.1). However, these terms did not exist in the common GO catagories of *Z. marina* and *P. yezoensis* (Figure 5.2). It was interesting that Go Slim terms of “cell proliferation (GO: 0008283)” and “death” (GO: 0016265) were specific to *Z. marina* (Figure 5.3).

**Figure 5:**
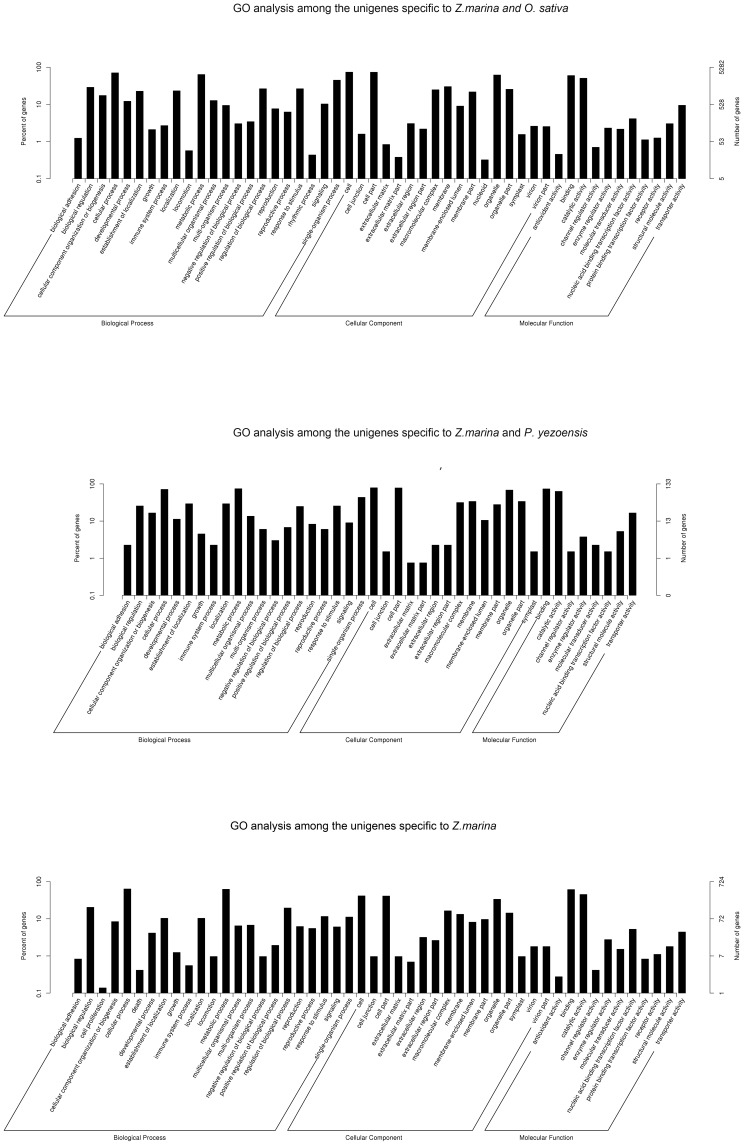
GO comparison for the specific unigenes among the *Z. marina* and *P. yezoensis* and *O. sativa*.

### Identification of *Z. marina* photosensory receptors

To gain insight on the information about the mechanism of *Z. marina* light sensing, we searched the transcriptome dataset for photoreceptor proteins and photoreceptor protein transcription factors (TF) that have been reported in land plants. After alignment with the known photoreceptor proteins, 6 unigenes were annotated as homologous to the photoreceptor proteins across different databases (Nt, Nr, GO, KEGG, Pfam and Prot). Among them, 2 unigenes homologous with red/far-red light photoreceptor proteins (phytochrome) were identified including comp17809_c0 to PhyA and comp10165_c0 to PhyB. 4 unigenes (comp8242_c0, comp12803_c0, comp5503_c0, and comp7891_c0) homologous with four blue light photoreceptors, such as cryptochrome 1, phototropin 1, phototropin 2-like, and PHR2-like, were also discovered. In addition, 89 unigenes related to transcriptional factors regulating phytochrome and cryptochrome were confirmed to exist in *Z. marina*. Seventy-three of these unigenes were related to transcriptional factors regulating phytochrome proteins including AUX/IAA, CO, COG1, COP1, COP9, DET1, EID1, ELF3, ELF4-like, FAR1, GBF, GI, GT-1, HY5, JAR1, PAT1, PIF3, PRR7, and SPA1. Thirty-one unigenes related to transcriptional factors regulating the blue photoreceptor proteins were identified including CDF1, CO, COP1, ELF3, FT, GI, HY5, and SPA1 (Table S2).

### Analysis of Candidate Ion Transport Genes

Ion channels and transporters are important regulators responding to osmotic stress and ionic stress. We identified unigenes related to different channels and transporters that have been reported to regulate ions in known species. Greater attention was paid to transporters and channels for Na^+^ efflux, K^+^ uptake, Cl^−^ channels, and H^+^ pumping (Table S3). It is noteworthy that 44 unigenes associated with K^+^ channels or transporters made up the largest proportion of identified transporters in this research. In total, 28 unigenes were annotated as involved in K^+^ channels, including the Shaker family, the TPK family, the Kir family, and the NGCC family. Sixteen unigenes were annotated as related to K^+^ transporters, such as the KUP/HAK/KT family. Nine unigenes involved in Na^+^ influx/efflux were identified, including 4 unigenes related to Vacuolar Na^+^/H^+^ antiporter (NHX1), 2 unigenes related to Na^+^/H^+^ antiporter (NHA1) and 3 unigenes related to sodium/calcium exchanger, respectively. SOS1 (plasma membrane Na^+^/H^+^) and SOS3 (Ca^2+^ binding protein) were included. Thirteen unigenes were annotated to Cl^−^ channels including the CLC (voltage gated chloride channel) family and the CCC (cation-chloride cotransporter) family with 10 unigenes and 3 unigenes, respectively. The unigenes associated with H^+^-ATPase were also identified, which may function in Na^+^ extrusion. Among 26 unigenes encoding the H^+^-ATPase family, the numbers of unigenes encoding vacuolar H^+^-ATPases (V-H^+^-ATPases), H^+^-pyrophosphatases (V-H^+^-PPases), and plasma membrane H+-ATPases (PM-H^+^-ATPases) were 19, 5, and 2, respectively. The presence of these ion channels, transporters, and pumps suggest putative functions in responding to the high ionic concentration of seawater.

## Discussion

For many non-model species without available genomic reference information, transcriptome sequencing is an effective and alternative method to gain insight on the information content of a genome. To date, several studies have published *Z. marina* transcriptomes via sequencing cDNA libraries and 454 pyrosequencing [3]–[7]. In this study, we obtained 24,216 unigenes using Illumina paired-end sequencing technology, after manipulating the environmental conditions to which the seagrass was exposed, including salinity, temperature, pH,light intensity, and light quality. The assembly result indicated that the mean length of unigenes was 1,006 bp and an N50 length of 1,802 bp, both of which are longer than any other *Zostera* transcriptome dataset. This indicates that our data could be effectively assembled. Furthermore, 27.29% of the transcripts had no hits against the other transcriptome data mentioned above, which shows that our experimental design is more suitable for identifying novel genes at the genomic level. Our data will extend the present transcriptome information of *Z. marina*. This study will also provide a foundation of identifying the functional genes, especially those specific to *Z. marina*, to further understand the mechanism of environmental adaptation and the evolutionary process of returning to the sea.

For gene annotation, the sequence similarity search was performed against protein databases including Nr, Swiss-Prot, GO, COG, and KEGG. In total, 64.3% of 24,216 unigenes were matched to known proteins in public databases. This implies that our Illumina paired-end sequencing generated a considerable portion of the *Z. marina* genes. Taking into account all BLAST hits in the NCBI NR protein database, the top ranked species with the most matched annotations was *G. max* (6.7%) followed by *V. vinifera* (6.6%), both of which are dicotyledons. This phenomenon agrees with our analysis of the ESTs from the full-length cDNA library [7]. However, this result was not expected as *Z. marina* is a monocotyledon and would be expected to be more homologous to *O. sativa* and *Z. mays*. This may indicate that the divergence of dicotyledons and monocotyledons occurred after *Z. marina* became established as a marine plant.

In our research, the plants were exposed to different environmental factors so the transcripts expressed in *Z. marina* leaves were mostly associated with adaptation to stress/defense. A large number of unigenes were assigned to a wide range of GO categories which revealed that the number of unigenes involved in response to stimulus and cell signaling were 3,742 and 1,508, respectively. With regards to KEGG pathways, particular attention was also given to environmental adaptation. The unigenes related to plant hormone pathways and signal transduction ranked near the top. Many studies have reported that plant adaptation to environmental stresses is dependent upon the activation of cascades of molecular networks involved in stress perception, signal transduction, and expression of specific stress-related genes and metabolites, such as plant hormones [34]–[36].

Light plays a major signaling role in plant development. Plants sense the quality (wavelength), intensity, duration (including day length), and direction of light via different photoreceptors. Plants have at least five types of sensory photoreceptors which allow them to accurately perceive the light environment and trigger responses that optimize photosynthesis and prevent damage: the blue light-sensing cryptochromes, phototropins and Zeitlupe family members, the red/far-red light-sensing phytochromes, and the UVB photoreceptor UVR8 (UV resistance locus 8) [37]–[39]. These photoreceptor families have been discovered with variable members in all higher plants tested. The phytochrome (PHY) family of receptors plays a major role in perceiving red (R) and far-red (FR) light. The *Arabidopsis* genome encodes five phytochromes (phyA–phyE) that have arisen through a series of gene duplications [40]. Monocots, such as rice and maize, contain only three members known as *PHYA*, *PHYB*, and *PHYC* [41]. It has been suggested that the functions of monocot and dicot phytochromes may not be totally conserved [42]. In this research, we only identified the unigenes homologous to *PHYA* and *PHYB* and none to *PHYC*. However, *PHYC* is considered to be widely distributed in flowering plants, diverging from *PHYA* prior to the origin of angiosperms [43]. Cryptochromes are receptors for blue and ultraviolet (UV-A) light that share sequence similarity to DNA photolyases, yet they have no photolyase activity [44],[45]. The *Arabidopsis* genome encodes three cryptochrome genes *CRY1*, *CRY2*, and *CRY3* [38]. *CRY1* and *CRY2* are also distributed in monocots such as rice and wheat [46]. Cryptochromes have also been found in lower plants, for example, 2 have been found in moss and 5 in fern [47],[48]. In this study, we only identified *CRY1* in *Z. marina*. In addition, we also found the unigenes encoding other blue light receptors including phototropin 1, phototropin 2-like, and PHR2-like. Further analyses should be performed to confirm the existence of PHYC and CRY2 and the function of these identified photoreceptors in *Z. marina*.

Salt tolerance is a complex process that involves many metabolic pathways. Ion channels have long been known to participate in action potential generation in animal, plant, and algal cells [49]. In this paper, we focus on several groups of ion transporters and channels that might contribute to the adaptation of *Z. marina* to high salinity seawater. The first group includes the channel and transporter genes that regulate potassium. Maintenance of K^+^ supply is a crucial feature of salt tolerance [50]. The discovery of various K^+^ channel and transporters shows that *Z. marina* leaves possess high affinity K^+^ uptake capacity. It is noteworthy that HKT, a key factor in determining salt tolerance in various species, was not identified in this research [34]. Na^+^ and Cl^−^ are chaotropic and predominantly associated with the deleterious effect of salinity. The Na^+^/H^+^ exchanger at the plasma membrane participates in the homeostasis of Na^+^ between the roots and shoots of land plants. At present, SOS1, SOS2, and SOS3 are known to participate in Na^+^/H^+^ exchanging in the plasma membrane. We also identified SOS1 in Illumina-generated transcriptome. SOS3, a myristoylated calcium binding protein, was discovered in 454 pyrosequencing data. SOS2, the serine/threonine protein kinase, can activate SOS1 and increase Na^+^/H^+^ exchange activity [51]. However, it was not identified in any dataset of the transcriptome. NHX-type antiporters are involved in Na^+^ extrusion from the cytosol into the apoplast, endosomal compartments, or vacuoles to avert the toxic effects of Na^+^ [52]. The compartmentation of Na^+^ ions into vacuoles, mediated by the NHX family, is driven by the vacuolar H^+^-translocating enzymes H^+^-ATPase and H^+^-PPase [53]. Seagrasses have been found to harbor a similar ratio of cellular Na^+^ to K^+^ as compared to terrestrial angiosperm species. H^+^-ATPase and the Na^+^/H^+^ antiporter play important roles in maintaining a low Na^+^/K^+^ ratio (a low Na^+^ concentration) within seagrass cells and allow the plant to thrive in seawater. The H^+^-ATPase generates an electrochemical membrane potential in the plasma membrane and Na^+^/H^+^ antiporters may transport Na^+^ from the interior to the exterior of the cell via this H^+^ gradient [54]. In this research, the many unigenes related to the NHX family and H^+^ pump indicate that Na^+^ compartmentation is an important mechanism for osmotic adjustment and Na^+^ detoxification in the cytosol of *Z. marina*. In addition to Na^+^, Cl^−^ compartmentation is also important for salt tolerance, and is performed by the Cl^−^ channel. In plants, the CLC family is the main channel mediating Cl^−^ sequestration in vacuoles [55]. We also identified the unigenes associated with the CLC family and this shows that *Z. marina* possesses a complicated and comprehensive salt tolerance system, which evolved as an adaptation to life in seawater.

## Conclusions

Using next generation sequencing technology, we produced a transcriptome profile for a seagrass species (*Z. marina*). We identified a substantial number of novel transcripts, representing a significant contribution to the accumulation of transcript data for *Z. marina*. In addition, we also identified potential transcripts for photoreceptors and related transcription factors, ion transporters and channels, which confirmed that NGS can serve as an alternative method for gene discovery. Importantly, our data shed light on *Z. marina* mechanisms used for adaptation to the sea water environment and improve our understanding of seagrass evolution.

## Supporting Information Legends

Table S1 **Distribution of** ***Z. marina*** **among KEGG pathways.**
(XLS)

Table S2 **The summary of unigenes related to transcriptional factors regulating the blue photoreceptor proteins in** ***Z. marina*** **.**
(XLSX)

Table S3 **The summary of unigenes related to transporters and channels in** ***Z. marina*** **.**
(XLSX)
